# Improving the Quality of Lateral Wrist Radiographs: A Closed-Loop Audit of Radiographic Compliance With Standard Positioning Criteria

**DOI:** 10.7759/cureus.100459

**Published:** 2025-12-30

**Authors:** Kunjan Barot, Adam khan Rahim, Izza Afzal, Hassan Imtiaz, Georgios Kouklidis, Talha Ahmed, Atizaz A Jan, Abdullah Durrani, Rizwana Monzur, John Lynch

**Affiliations:** 1 Trauma and Orthopaedics, University Hospitals Dorset, Poole, GBR; 2 Internal Medicine, Hayatabad Medical Complex, Peshawar, PAK

**Keywords:** clinical audit, general radiology, hand-wrist radiograph, orthopaedics trauma, quality improvement (qi)

## Abstract

Background

Wrist injuries represent a substantial proportion of acute musculoskeletal presentations, with distal radius and carpal fractures among the most frequently encountered upper-limb fractures in emergency and orthopaedic services. Accurate lateral wrist radiographs are essential for detecting osseous and carpal alignment abnormalities, guiding management, and preventing complications. However, radiographic quality is highly dependent on adherence to established positioning standards. Inadequate positioning may obscure key anatomical relationships, contributing to diagnostic errors, treatment delays, unnecessary imaging escalation, and increased medico-legal vulnerability. This closed-loop audit aimed to evaluate baseline compliance with recognised radiographic standards for lateral wrist radiographs, implement targeted educational interventions, and reassess compliance to determine the effectiveness of these measures.

Methods

A retrospective audit was conducted at Poole District General Hospital, United Kingdom. Adult patients aged 18-65 years who underwent lateral wrist radiography were evaluated across two cycles (50 radiographs per cycle). Radiographs were reviewed independently by two senior registrars and assessed against three standards: superimposition of radial and ulnar styloid processes, inclusion of the distal radius/ulna, all carpal bones, and proximal metacarpals, and demonstration of the piso-scapho-capitate relationship. Educational interventions included posters, teaching sessions, and dissemination of positioning guidelines. Chi-square testing analysed differences between audit cycles, with a p-value of less than 0.05 considered statistically significant.

Results

A total of 50 radiographs were evaluated in each audit cycle. In the first cycle, compliance with Standard 1 (superimposition of the radial and ulnar styloid processes) was 72% (n=36/50), Standard 2 (appropriate anatomical inclusion) was 98% (n=49/50), and Standard 3 (piso-scapho-capitate relationship) was 50% (n=25/50). Following targeted educational interventions, compliance in the second cycle improved to 92% (n=46/50) for Standard 1, 100% (n=50/50) for Standard 2, and 82% (n=41/50) for Standard 3. Improvements were statistically significant for Standard 1 (p=0.047) and Standard 3 (p=0.014), while Standard 2 remained consistently high across both cycles (p=0.992). Overall, compliance across all standards increased substantially between cycles, demonstrating the clear effectiveness of the educational interventions in achieving and surpassing the 80% compliance target.

Conclusions

Simple, targeted educational interventions significantly improved adherence to radiographic positioning standards. Enhanced radiograph quality supports accurate diagnosis and clinical decision-making. Sustained improvement will require ongoing education and periodic re-audit, reinforcing high-quality imaging as a cornerstone of safe and efficient musculoskeletal care.

## Introduction

Wrist injuries constitute a substantial proportion of acute presentations to emergency and orthopaedic services worldwide. In the United Kingdom, wrist fractures represent a high-volume workload for the National Health Service (NHS) due to their frequency and the ageing demographic [1]. Distal radius fractures alone account for up to 18% of all fractures among older adults [2] and are also prevalent among younger individuals sustaining high-energy trauma [3]. Epidemiological studies from diverse healthcare systems similarly demonstrate a rising incidence of distal radius and carpal injuries, underscoring their public health significance [4,5].

Accurate imaging plays a pivotal role in diagnosing wrist fractures. The wrist joint is a complex condyloid articulation composed of the distal radius and the proximal carpal row (excluding the pisiform), forming a biomechanically intricate structure requiring precise radiographic visualisation [6]. Because clinical examination alone frequently fails to localise carpal pathology, wrist radiographs, particularly the lateral view, are indispensable for both diagnosis and treatment planning.

Radiographic quality varies considerably depending on patient positioning, anatomical inclusion, and the radiographer’s technical experience. Clark’s Positioning in Radiography remains the internationally recognised reference standard for optimal wrist imaging [7]. For lateral wrist radiographs to be diagnostically valuable, they must demonstrate: superimposition of the radial and ulnar styloid processes, full inclusion of relevant osseous structures (distal 1/3 of radius/ulna, all carpal bones, and proximal metacarpals), and maintenance of the piso-scapho-capitate relationship where the pisiform overlies the central third between the distal scaphoid pole and capitate [8,9].

These parameters are not arbitrary; poor radiographic technique can lead to misinterpretation, missed injuries, inappropriate management, and significant morbidity. Distal radius fractures, if inaccurately assessed, are associated with complications such as median nerve neuropathy, tendon ruptures, and malunion [10]. Delay in operative treatment beyond two weeks has been linked with inferior functional outcomes [11]. Similarly, scaphoid fractures, often subtle and best appreciated on the lateral view, carry a high risk of nonunion, malunion, and degenerative wrist collapse when missed [12-15].

Given the diagnostic significance of lateral wrist imaging and the medico-legal risks associated with error [16], optimising radiographic accuracy is essential. Several studies have emphasised the value of system-level interventions such as radiographer education, imaging workflow optimisation, and audit cycles to improve the quality of musculoskeletal imaging [17]. However, few audits specifically address adherence to lateral wrist radiograph standards in UK district hospitals.

The aim of this closed-loop audit was therefore to assess baseline compliance with the three established lateral wrist radiograph standards, implement targeted educational interventions, and re-evaluate compliance to determine whether simple, cost-effective measures can produce improvements in radiographic practice.

## Materials and methods

This retrospective closed-loop audit was conducted at Poole Hospital, a district general hospital in the United Kingdom. The audit was registered with the clinical governance department on Nov 14, 2025 (Audit registration number: 365-2526). All adult patients aged 18-65 years who underwent lateral wrist radiography between January-March 2025 (Cycle 1) and September-November 2025 (Cycle 2) were eligible. Exclusion criteria included previous wrist surgery, pre-existing localised bony pathology, immobilisation in plaster, and age outside the predetermined range. Patients beyond 65 years of age were excluded due to the high likelihood of the presence of osteoarthritis, which could potentially distort the lateral radiograph view, and lead to inaccuracies in determining radiographic parameters. 

Radiographs were identified using the Picture Archiving and Communication System (PACS). Each lateral view was reviewed independently by two senior orthopaedic registrars on separate occasions to minimise observer bias. Discrepancies between radiographic parameter assessments were referred to a third reviewer (consultant musculoskeletal radiologist). 

Radiographs were assessed against three standards derived from Clark’s Positioning in Radiography [8], namely: Superimposition of radial and ulnar styloid processes, Inclusion of distal 1/3 of radius and ulna, all carpal bones, and proximal 2/3 of metacarpals, and demonstration of the piso-scapho-capitate relationship. A target compliance of ≥80% for each standard was agreed in consultation with a senior hand and wrist surgeon. Audit standards are presented in Table 1.

**Table 1:** Audit Standards.

Audit Standard(s)	Target Compliance (%)
Superimposition of the radial and ulnar styloid processes	80%
Inclusion of the distal 1/3rd of all meta-carpal bones, all the carpal bones and the distal 1/3rd of radius and ulna	80%
Demonstration of the Piso-Scapho-capitate relationship.	80%

Following cycle 1, educational interventions were implemented, including a poster detailing proper positioning techniques displayed in the radiology department and trauma meeting rooms (Appendix 1). A formal teaching session delivered by an associate specialist hand surgeon (Appendix 2) to increase awareness regarding appropriate positioning during radiographic imaging was undertaken. This session was delivered in person to radiographers and orthopedic doctors. The subsequent second audit cycle was undertaken following the implementation of these changes to evaluate their impact on radiographic practice. 

The clinical audit was registered with the local Clinical Governance department, and based on parameters being evaluated and the fact that this was intended to be a clinical audit, ethical approval was not deemed necessary. Data was collected over a Microsoft Excel spreadsheet, stored on the trust's intranet. Data analysis was performed using JASP software (JASP, version 0.18.3; JASP Team). Patient identifiers were removed prior to analysis. Chi-square testing was applied to compare compliance between cycles, with statistical significance defined as p< 0.05. The degrees of freedom used during statistical testing were 1. Phi-coefficient (φ) was used to quantify effect size.

## Results

Fifty radiographs met the inclusion criteria for the first cycle. Compliance rates were: Standard 1: 72% (n=36), Standard 2: 98% (n=49), Standard 3: 50% (n=25). Radiographs met the target compliance (80%) only against Standard 2, but fell short with regard to the remaining standards. Following the implementation of the educational intervention, the second audit cycle was conducted, with 50 radiographs meeting the inclusion criteria during this period. Observed compliance improved in all three parameters; Standard 1: 92% (n=46, χ²(1)=3.93, df=1, φ=0.20, p=0.047), Standard 2: 100% (n=50, χ²(1)=0.0001, df=1, φ=0.00, p=0.992), Standard 3: 82% (n=41, χ²(1)=6.03, df=1, φ=0.25, p=0.014). All radiographs therefore met the target compliance (80% or above) with respect to all three set standards. Statistically significant improvements were achieved for Standards 1 and 3, with no significant change for Standard 2, which was already near optimal, thereby signifying a ceiling effect. No discrepancies were observed between the evaluation of radiographic parameters by the two independent assessors, for both audit cycles, and therefore none of the cases required intervention from a third reviewer. The comparison of results between the audit cycles with statistical analysis is given in Table 2.

**Table 2:** Comparison between audit cycles, with statistical analysis *p-value <0.05 set as cut-off for statistical significance

Standard (s)	Observed Compliance Cycle 1 (%, n)	Observed Compliance Cycle 2 (%, n)	Degrees of freedom	Chi-square value	Phi-coefficient (φ)	*p-value
Superimposition of the radial and ulnar styloid processes	72% (n=36)	92% (n=46)	1	3.93	0.20	0.047
Inclusion of the distal 1/3rd of all meta-carpal bones, all the carpal bones and the distal 1/3rd of radius and ulna	98% (n=49)	100% (n=50)	1	0.0001	0.0	0.992
Demonstration of the Piso-Scapho-capitate relationship.	50% (n=25)	82% (n=41)	1	6.03	0.25	0.014

## Discussion

This audit demonstrates how improvements in radiographic quality can be achieved following simple yet effective targeted educational interventions. Accurate lateral wrist radiographs play a central role in diagnosing distal radius and carpal injuries, which are among the most common fractures worldwide [1-5]. High-quality radiographs reduce diagnostic uncertainty, enhance management planning, and prevent adverse outcomes.

The initial compliance rates in cycle 1, particularly for Standards 1 (72%) and 3 (50%), were comparable to findings in previous studies that report widespread variability in musculoskeletal radiographic technique [17,18]. Standard 2 demonstrated high baseline compliance (98%), consistent with literature suggesting that anatomical inclusion is generally easier to achieve than rotational accuracy [7].

Following the educational interventions, compliance improved significantly to 92% and 82% for Standards 1 and 3, with phi coefficients (0.20 and 0.25, respectively) representing a medium-level correlation. These improvements mirror the results of educational initiatives reported in radiology workflow optimisation literature, where even brief teaching interventions produced measurable enhancements in musculoskeletal imaging quality [17].

The piso-scapho-capitate relationship is the most technically demanding criterion, requiring precise rotational positioning. Its improvement from 50% to 82% is clinically important because the lateral view is critical for diagnosing scaphoid pathology. Missed scaphoid fractures have been shown to lead to nonunion, malunion, and long-term functional impairment [13-15]. Improved radiographic quality may reduce dependence on advanced imaging modalities such as MRI, which, although highly sensitive, carries significant cost and resource implications [16].

Furthermore, the improvements seen in this audit can potentially have meaningful medico-legal benefits. Hand and wrist injuries account for a large proportion of malpractice claims in orthopaedics, often relating to missed fractures or inadequate imaging documentation [16]. Enhanced radiographic standards, therefore, contribute not only to clinical excellence but also to risk mitigation. Improved radiograph quality can potentially reduce unnecessary CT and MRI utilisation, aligning with best-practice imaging algorithms that emphasise judicious resource use in low- and high-resource settings [17,18].

The limitations of this study include a small sample size of 50 cases, which limits statistical power. Given this was a single-centre study, the results are reflective of local practice and thus limit the generalisability. The radiographic technique being evaluated lends itself to a large amount of variability amongst radiographers, and this operator variability serves as a confounding factor, limiting the objectivity of the results. The study examines technical compliance, but does not relate this to diagnostic outcomes, specifically if any pathologies were missed due to inadequate radiographic techniques; this could reinforce the recommendations to aim for higher compliance with recommended radiological parameters while performing lateral wrist radiographs. Lastly, the improvements observed also need to be evaluated for long-term sustainability, and a further evaluation at a later period in time would have allowed for a more robust conclusion to be derived for the proposed recommendations.

## Conclusions

This closed-loop audit demonstrates that simple, low-cost educational interventions can yield significant improvements in lateral wrist radiograph quality. Following targeted teaching and visual prompts, compliance with all three radiographic standards increased, achieving or exceeding the 80% target. High-quality radiographs are essential for accurate diagnosis, optimal management of wrist injuries, reduction in unnecessary advanced imaging, and protection against medico-legal risk.

To maintain these improvements, regular audit cycles, ongoing education, and multidisciplinary collaboration are essential. Embedding these radiographic standards into routine clinical practice will enhance patient safety, diagnostic precision, and healthcare efficiency.

## Figures and Tables

**Figure 1:**
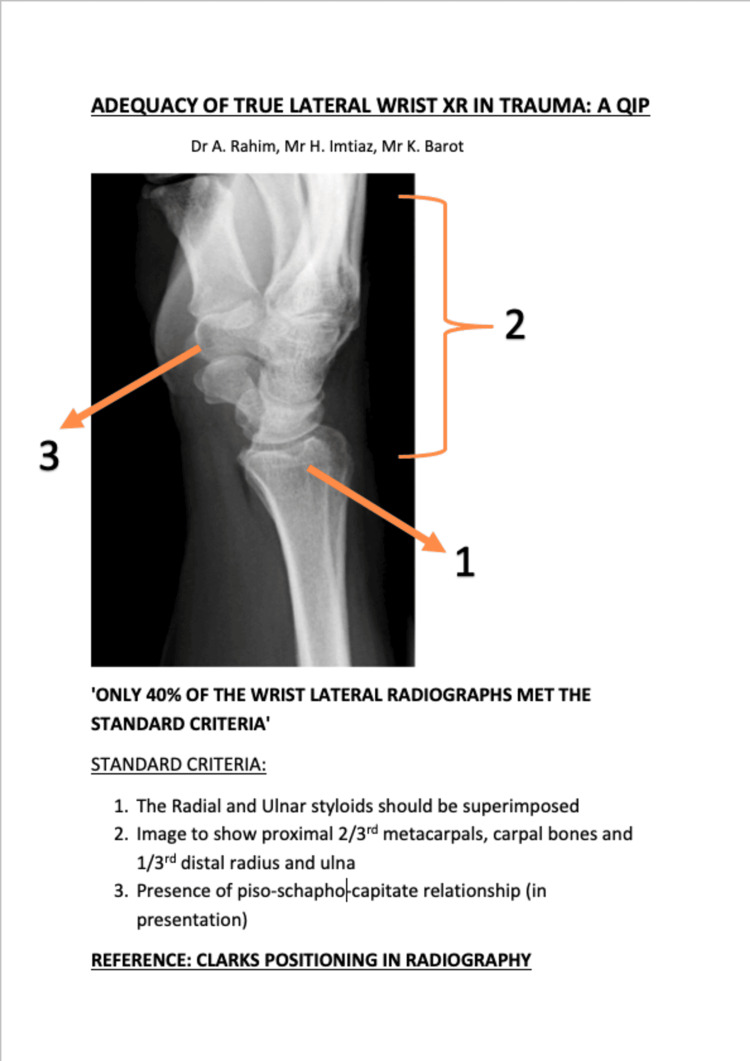
Poster Poster displayed to raise awareness of adequate lateral wrist radiograph imaging. These were displayed in the radiology department and trauma meeting rooms

**Figure 2:**
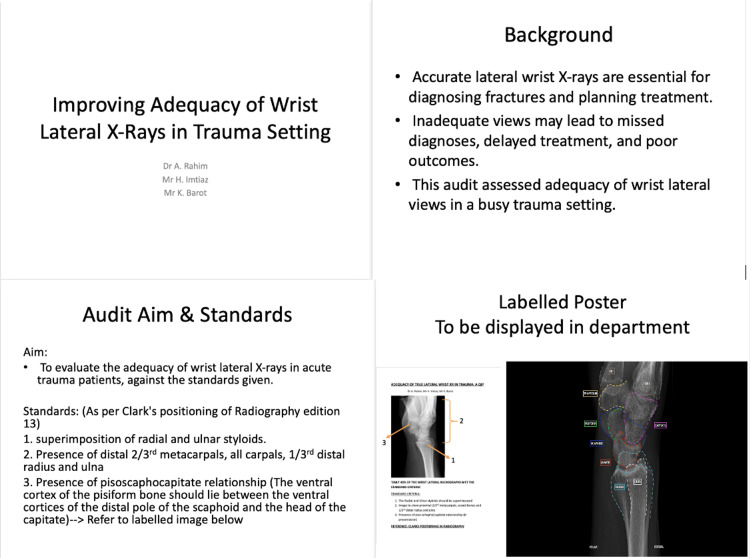
Presentation (Slides 1-4) Powerpoint presentation (10 slides) delivered by a Senior Hand and Wrist Surgeon to Radiographers and Orthopaedics team doctors, to increase awareness of radiographic parameter to look for when obtaining lateral wrist radiographs.

**Figure 3:**
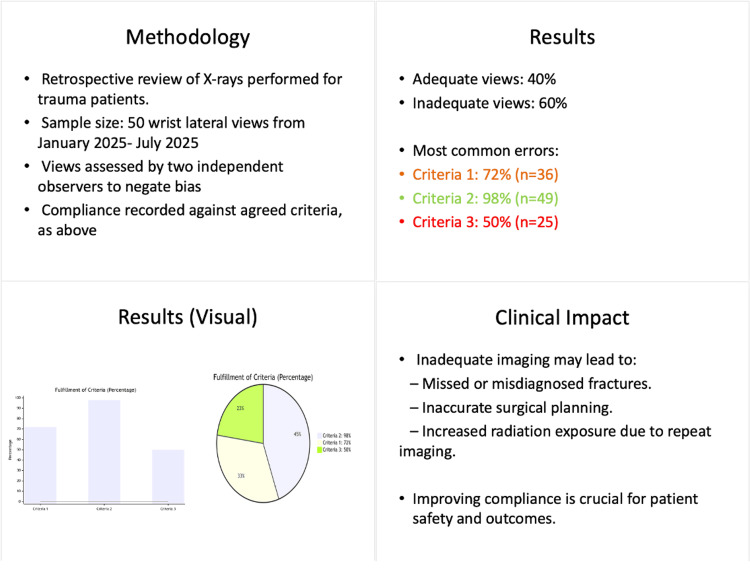
Presenation (Slides 5-8) Powerpoint presentation (10 slides) delivered by a Senior Hand and Wrist Surgeon to Radiographers and Orthopaedics team doctors, to increase awareness of radiographic parameter to look for when obtaining lateral wrist radiographs.

**Figure 4:**
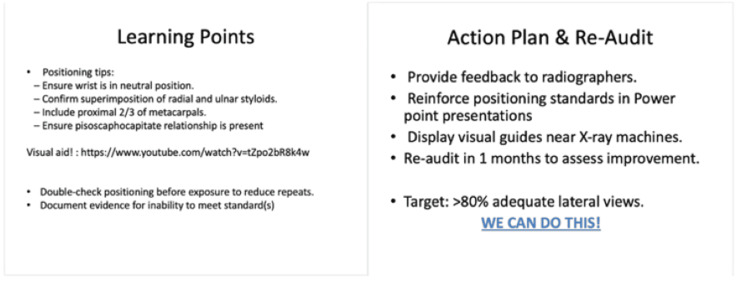
Presentation (Slides 9-10) Powerpoint presentation (10 slides) delivered by a Senior Hand and Wrist Surgeon to Radiographers and Orthopaedics team doctors, to increase awareness of radiographic parameter to look for when obtaining lateral wrist radiographs.

## Appendices

Appendix 1 (Poster)

Figure 1 represents the poster used.

Appendix 2 (Presentation)

Figures 2-4 depict presentation slides.
